# The Role of Cyclic Nucleotide Signaling Pathways in Cancer: Targets for Prevention and Treatment

**DOI:** 10.3390/cancers6010436

**Published:** 2014-02-26

**Authors:** Alexandra M. Fajardo, Gary A. Piazza, Heather N. Tinsley

**Affiliations:** 1 Drug Discovery Research Center, Mitchell Cancer Institute, University of South Alabama, 1660 Springhill Ave, Suite 3029, Mobile, AL 36604, USA; E-Mails: afajardo@health.southalabama.edu (A.M.F.); gpiazza@health.southalabama.edu (G.A.P.); 2 Department of Biology, Chemistry, and Mathematics, University of Montevallo, Station 6480, Montevallo, AL 35115, USA

**Keywords:** cyclic nucleotides, cAMP, cGMP, cyclic nucleotide phosphodiesterases, protein kinases, PKG, PKA

## Abstract

For more than four decades, the cyclic nucleotides cyclic AMP (cAMP) and cyclic GMP (cGMP) have been recognized as important signaling molecules within cells. Under normal physiological conditions, cyclic nucleotides regulate a myriad of biological processes such as cell growth and adhesion, energy homeostasis, neuronal signaling, and muscle relaxation. In addition, altered cyclic nucleotide signaling has been observed in a number of pathophysiological conditions, including cancer. While the distinct molecular alterations responsible for these effects vary depending on the specific cancer type, several studies have demonstrated that activation of cyclic nucleotide signaling through one of three mechanisms—induction of cyclic nucleotide synthesis, inhibition of cyclic nucleotide degradation, or activation of cyclic nucleotide receptors—is sufficient to inhibit proliferation and activate apoptosis in many types of cancer cells. These findings suggest that targeting cyclic nucleotide signaling can provide a strategy for the discovery of novel agents for the prevention and/or treatment of selected cancers.

## 1. The Physiology of Cyclic Nucleotide Signaling

**Figure 1:**
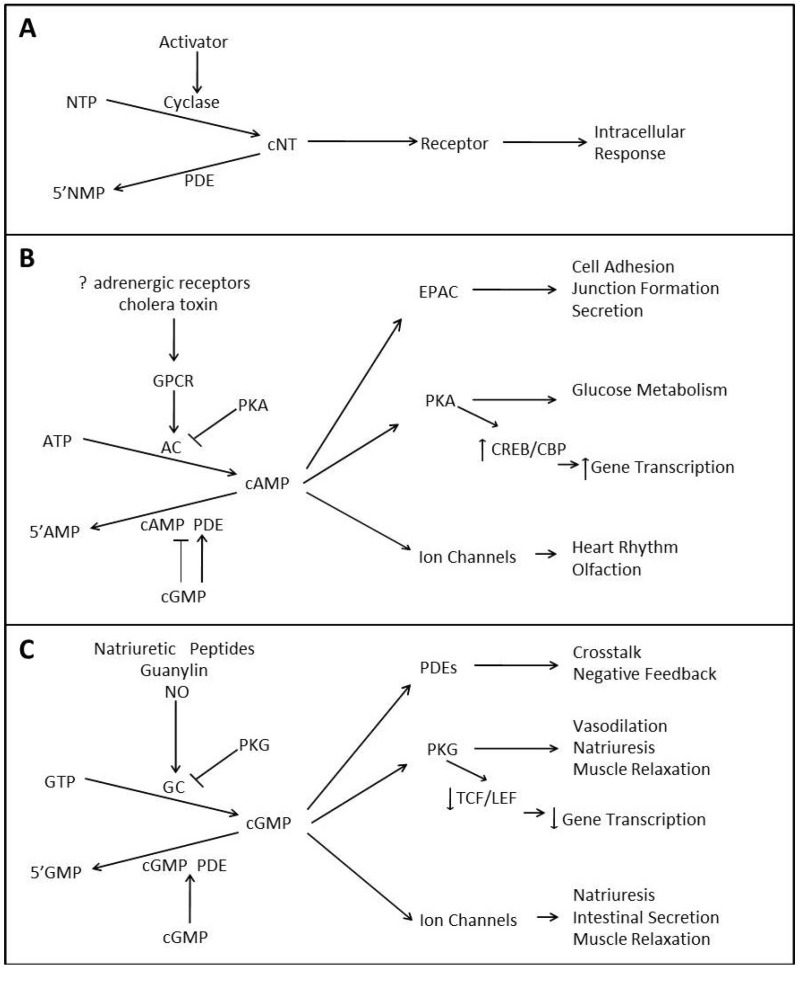
Generalized illustrations of cyclic nucleotide signaling (**A**); cAMP signaling (**B**); and cGMP (**C**). A, demonstrates cyclic nucleotide (cNT) production through nucleotide precursor (NTP) and activation of selective cyclase and activation receptor and intracellular response or degradation of cNT through phosphodiesterase (PDE) activity. B, production of cAMP by adenylyl cyclase (AC) can be regulated by GPCR and PKA. cAMP signaling can lead to the activation of PKA, EPAC and ion channels. Cyclic AMP can also be degraded by PDEs (PDE1, 2, 3, 4, 7, 8, 10, or 11) and cGMP can inhibit selective PDE cAMP degrading activity. C, production of cGMP by guanylyl cyclase (GC) can be regulated by nitric oxide (NO) and PKG. cGMP signaling can lead to the regulation of other PDEs, PKG and ion channels. Cyclic GMP can also be degraded by PDEs (PDE1, 2, 3, 5, 6, 9, 10, or 11).

The cyclic nucleotides, cyclic adenosine monophosphate (cAMP) and cyclic guanosine monophosphate (cGMP), have long been recognized as important intracellular signal transduction molecules, acting as second messengers between an extracellular signal such as a hormone, neurotransmitter, or cytokine and the elicited intracellular response. While the specific function of a given signal varies according to the cell type, extracellular environment, stimulus activating the signal, localization of the signal, and the type of cyclic nucleotide formed, as depicted in Figure 1A, an extracellular signal will generally activate a cyclase enzyme, which catalyzes the formation of the cyclic nucleotide (cNT) from its nucleotide triphosphate precursor (NTP). 

Once formed, the cyclic nucleotide will affect the activity of downstream effector molecules including kinases, ion channels, transcription factors, and scaffolding proteins. Both the amplitude and duration of a cyclic nucleotide signal also varies and is largely dependent on the expression and activity levels of cyclic nucleotide phosphodiesterase (PDE) enzymes, which catalyze the hydrolytic breakdown of cyclic nucleotides.

### 1.1. cAMP Signaling

First described in the late 1950s, cAMP is the more well studied of the cyclic nucleotides. The intracellular level of the second messenger, cAMP, is regulated by opposite activities of two enzymes, adenylyl cyclase (ACs) and PDEs [1,2]. Both ACs and PDEs are regulated by numerous signaling pathways including, calcium signaling through calmodulin (CaM) and calcineurin, G-proteins, inositol lipids (e.g., PKC) and receptor tyrosine kinases [2]. As reviewed previously [3] and depicted in Figure 1B, cAMP is produced from its precursor, ATP, through the catalytic activity of the ACs. Differing primarily in tissue distribution and subcellular localization, nine membrane-bound and one soluble AC have been identified in mammals [4]. The majority of ACs are indirectly activated by various stimuli including adrenergic agonists, which bind to G protein coupled receptors (GPCRs) on the cell membrane resulting in the activation of the GPCR and release of the Gα_s_ subunit that is subsequently responsible for binding to and activating AC, thus stimulating the production of cAMP [5,6]. The balance of cAMP signaling is essential to multiple cellular processes, including immune function, growth, differentiation, gene expression and metabolism [6].

In 1983, Buxton and Brunton [7], demonstrated how different GPCR agonists could stimulate, in a compartmentalized manner, equivalent increases in cAMP levels but yet result in receptor specific-mediated outcomes in cardiomyocytes [6,7]. Concentrations of cAMP can be regulated by processes throughout the whole cell and within membrane regions. The most recently identified factors contributing to cAMP compartmentalization are the ATP-binding cassette (ABC) transporters. ABC transporters function as ATP-dependent transporters of multiple endogenous and exogenous substances across extracellular and intracellular membranes. Two of the members of the ABC transporter family, multidrug resistance protein 4 (MRP4; also known as ABCC4) and MRP5 (also known as ABCC5) have both demonstrated an energy dependent export of cNTs. Both cAMP and cGMP were among the first substrates identified for MRP4. Although the whole cell intracellular levels of these cyclic nucleotides remained unchanged with increased MRP4 activity, MRP4 was responsible for their regulation at the microdomain level [8]. MRP4 promotes the modulation of localized membrane cAMP concentrations which are coupled to GPCR-mediated events. To accomplish this local regulation of cAMP, MRP4 forms macromolecular complexes in specialized subcellular domains [9]. Similar to MRP4, MRP5 can also efflux both cGMP and cAMP, thus reducing their intracellular availability [10]. Both MRP4 and MRP5 may function as cNT overflow pumps thus regulating cNT levels when there is an overproduction of cNTs or inhibited PDE activity [9].

The regulation of cAMP levels can be inhibited by GPCR subunits which modulate the activity of ACs in some cell types and cAMP-dependent serine/threonine protein kinase (PKA) activity can act as a negative regulator of cAMP signaling by phosphorylating and inactivating ACs [3], the amplitude and duration of a cAMP signal within most cells is largely dependent on PDE enzyme activity, which is responsible for hydrolyzing cAMP to 5'AMP in order to terminate its signal [11]. To date, eleven PDE isozyme families consisting of 24 distinct genes have been identified. Due to alternative splicing and post-translational modifications, each PDE gene is capable of producing multiple protein products within a single cell or tissue whereby up to 100 isoforms are believed to exist in the human proteome. Each PDE isozyme family displays unique biochemical properties including substrate specificity, regulatory processes, and/or pharmacological sensitivity, as summarized in Table 1 [12,13,14,15,16,17]. Hydrolysis of cAMP is primarily mediated by the PDE1, 2, 3, 4, 7, 8, 10, and 11 isozyme families, with PDE4, 7, and 8 being cAMP selective and therefore generally unable to hydrolyze cGMP under physiological conditions [18]. Despite relatively equal levels of AC and cAMP PDE expression in most cell types, the rate of cAMP hydrolysis in virtually all human tissues far exceeds the rate of synthesis, making PDE enzymes an important determinant of intracellular cAMP levels, which, under basal conditions, are typically less than 5 pmol per mg of protein [19].

**Table 1:** Human PDE isozymes are divided into 11 families and differ according to substrate specificity, mechanisms of regulation, and sensitivity to inhibitors [12,13,14,15,16,17,20,21]. ↑ represents an increase in catalytic activity, whereas ↓ represents a decrease in catalytic activity. * Number of isozymes refers to the number of distinct protein products derived from all genes within a given family that have been identified to date.

Isozyme Family	Number of Genes	Putative Number of Isozymes *	Substrate Specificity	Regulators	Inhibitors
1	3	21	dual	Ca^2+^-CaM: ↑	IC224, SH51866,
				PKA: ↓	8-methoxymethyl-IBMX
2	1	3	dual	cGMP: ↑	EHNA, BAY 60-7550, PDP, IC933
3	2	4	dual	cGMP:↓	Milrinone, Tolafentrine, Cilostazol, Cilostamide, OPC-33540
				PKA: ↑	
4	4	31	cAMP	PKA: ↓	Rolipram, Cilomilast, Roflumilast, Ro20-1724, Denbufylline, AWD12281
5	1	3	cGMP	cGMP: ↑	Sildenafil, Zaprinast,
				PKG: ↑	Dipyridamole, Tadalafil, Vardenafil, DMPPO, E402, DA8159, 8-methoxymethyl-IBMX
6	3	3	cGMP	Transducin: ↑	Sildenafil,
					Dipyridamole, Zaprinast
7	2	7	cAMP	unknown	BRL 50481, IC242, Dipyridamole, Thiadiazoles
8	2	9	cAMP	unknown	Dipyridamole
9	1	2	cGMP	unknown	BAY73-669, SCH 51886, Zaprinast
10	1	10	dual	PKA: ↑	Papaverine, PF-2545920, PQ-10, Dipyridamole
11	1	4	dual	unknown	BC 11-38, Dipyridamole

The cellular effect of a given cAMP signal depends largely on the specific receptor(s) that is activated, which is most often dictated by the cell type and/or subcellular localization of the signal. Intracellular receptors for cAMP include the guanine nucleotide exchange factor (GEF) exchange protein activated by cAMP (EPAC), ion channels such as the cyclic nucleotide gated (CNG) channels and the hyperpolarization-activated cyclic nucleotide gated (HCN) channels, and PKA, EPAC, a GEF, that activates the small G protein Rap1 in order to mediate cell adhesion, junction formation, and secretion [2,3]. EPAC can mediate cAMP anti-apoptotic and pro-apoptotic signaling through a PKA-independent mechanism in a cell-type specific manner [22].

Responsible for mediating heart rate and rhythm, HCN channels are regulated by the binding of cAMP, which shifts the membrane potential necessary for the channels to open [19]. CNG channels, which are important mediators of olfaction, are also regulated by the binding of cAMP but in a manner independent of membrane potential [3]. The holoenzyme, PKA, in its inactive form is a tetramer consisting of two regulatory subunits (R) and two catalytic subunits (C). Both R subunits contain two binding sites for cAMP. Upon binding of cAMP the two C subunits are released and activated to phosphorylate their substrates [1]. PKA phosphorylates metabolic enzymes to mediate glucose metabolism and the transcription factor cAMP response element binding protein (CREB) to promote gene transcription [3,19]. There are four PKA regulatory subunits (RIα, RIIα, RIβ and RIIβ) and three catalytic subunits (Cα, Cβ, and Cγ). The regulatory subunits are differentially expressed in various cell types and subcellular localizations [23,24]. The catalytic subunits can be combined with the four regulatory isoforms to obtain enzymes with different biochemical properties [23]. For instance, PKA-RI has been found primarily in the cytosol and more can be more readily dissociated by cAMP binding than PKA-RII [24]. Within the same cell elevated cAMP levels and subsequent PKA activity by different agonists can lead to different physiological responses due to the cellular distribution of the isoforms their particular biochemical properties.

One of the key downstream activation pathways regulated by cAMP-PKA signaling is the activation of the transcription factor CREB. CREB is a 43 kDa basic/leucine zipper transcription factor that is highly conserved and expressed in most tissue types. The transcriptional activation of CREB is due to phosphorylation of serine 133 by a serine-threonine kinase (i.e*.*, PKA). This phosphorylation stimulates the interaction of CREB with its co-activator CREB-binding protein (CBP) [25]. CREB plays a critical role in regulating the expression of genes who promote oncogenesis [26]. The activation of CREB is not PKA specific, CREB phosphorylation can be stimulated by multiple kinases including AKT, pp90 ribosomal S6 kinase (p90Rsk), and calcium/calmodulin dependent kinases [26]. CREB regulates a multitude of genes and can be overexpressed and constitutively activated in many cancer types [26]. CREB regulates the expression of several genes involved in metabolism, signaling, proliferation, differentiation and survival [25].

### 1.2. cGMP Signaling

In a manner similar to cAMP and as depicted in Figure 1C, cGMP is formed through the activity of guanylyl cyclase (GC) enzymes from its precursor GTP. One distinct difference from cAMP signaling is that GC enzymes are more evenly dispersed between the membrane and the cytosol of cells and are directly activated by their stimuli [3]. Seven particulate or membrane bound GCs (pGC) have been identified, each consisting of a single transmembrane region [27]. As with the membrane-bound AC isoforms, pGC isoforms differ largely in their tissue distribution but also in their sensitivity to ligands, which include natriuretic peptides, small paracrine peptide hormones such as, guanylin, enterotoxins, and certain cytokines. Conversely, the heme-containing soluble GC (sGC) enzyme is restricted to the cytoplasm and is solely activated by nitric oxide (NO) under physiological conditions [3].

Similar to cAMP, cGMP levels are predominantly controlled by degradation via PDE enzymes. PDE1, 2, 3, 5, 6, 9, 10 and 11 families are capable of hydrolyzing cGMP with PDE5, 6, and 9 being selective for cGMP [11]. In most tissues, PDE5 is the isoform predominantly responsible for cGMP hydrolysis and subsequent termination of a cGMP signal. The activity of the PDE5 enzyme is tightly controlled by cGMP signaling. In the presence of a cGMP signal, cGMP will bind to the GAF A domain of the *N*-terminal region of the PDE5 protein to promote its phosphorylation at a separate *N*-terminal site by the cGMP-dependent serine/threonine protein kinase (PKG), an event that produces a several-fold increase in the activity of the enzyme while simultaneously increasing the affinity of the catalytic site for cGMP [28]. The regulation of PDE activity is unique to cGMP signaling and serves multiple functions within cells such as acting as negative feedback for cGMP signaling by activating cGMP specific PDE5 or acting as crosstalk between cyclic nucleotide pathways by increasing or decreasing the activity of non-selective PDE isozymes such as PDE2 or PDE3, respectively [11].

Some GC isoforms serve as substrates for phosphorylation by PKG resulting in decreased catalysis and, therefore, decreased formation of cGMP. However, similar to cAMP signaling, the specific effect of a cGMP signal depends largely on the receptor(s) that is activated. Under standard physiological concentrations, which for cGMP are typically tenfold less than those observed for cAMP [19], cGMP preferentially activates receptors distinctly different than those activated by cAMP, including certain PDE isozymes, PKG, and CNG ion channels [3]. Modulation of CNG channel activity is a more common event in cGMP signaling compared to cAMP signaling and serves as an important step for mediating the effects of cGMP on phototransduction, natriuresis, and intestinal fluid and electrolyte secretion [29]. Similar to PKA and cAMP signaling, PKG mediates cGMP signaling by modulating down-stream signaling effects [3]. For example, PKG can phosphorylate and activate myosin phosphatase to promote vasodilation and muscle relaxation [27].

A central mediator of cGMP signaling is PKG, which activated by cGMP binding and phosphorylates down-stream substrates [18]. PKG is a serine/threonine protein kinase which is highly versatile and plays a diverse role in regulating multiple cellular processes (i.e*.*, vasodilation, cell differentiation, cell proliferation and apoptosis). In humans there are three isoforms of PKG, the alternatively spliced α and β isoforms of type 1 PKG (PKGIα and PKGIβ) and type 2 PKG (PKGII) [30]. Both isoforms of PKGI are widely distributed, but vary in their tissue expression. PKGIα is found mainly in the lung, heart, platelets and cerebellum, while PKGIβ and PKGIα are highly expressed in the smooth muscles of the uterus, intestine and trachea [31]. PKGII is more restricted in its expression to the brain, intestine and kidney [30,32]. The down-stream substrates regulated by PKG include those involved in calcium homeostasis, platelet activation and adhesion, smooth muscle contraction, cardiac function, and gene expression [33].

One of the down-stream signaling events mediated by PKG is the regulation of β-catenin protein levels. Elevations in β-catenin expression promote interactions with the TCF/LEF transcription factors to activate growth-related target genes [34]. Studies suggest that upon cGMP increase, PKG is activated and directly phosphorylates β-catenin leading to increased proteasomal degradation and inhibition of TCF/LEF transcriptional regulation [35,36,37]. However, this mechanism has been disputed by recent evidence demonstrating activation of PKG leads to inhibition of β-catenin mRNA expression and implicates the transcription factor FOXO4 for competing with TCF for β-catenin binding [34]. Although the β-catenin/TCF signaling pathway plays a critical role in tumorigenesis especially in the colon, further studies are needed to determine the mechanism(s) of PKG inhibition of β-catenin/TCF/LEF signaling.

### 1.3. Crosstalk between Cyclic Nucleotide Signaling Pathways

An additional level of complexity arises when considering the crosstalk between cAMP and cGMP signaling pathways. The most notable source of crosstalk between the pathways is found in the ability of cGMP to modulate the activity of various PDEs, particularly PDE2 and PDE3, which can hydrolyze both cAMP and cGMP, but have higher affinity for cAMP. For example, low nanomolar concentrations of cGMP are sufficient to produce more than a tenfold increase in cAMP hydrolysis by PDE2 and all but complete inhibition of cAMP hydrolysis by PDE3 [28]. In fact, it is thought that the effects of atrial natriuretic peptide (ANP) on aldosterone secretion are at least partially due to a drop in cAMP levels that is mediated by cGMP signaling following the activation of PDE2 [11].

Phosphorylation events mediated by PKA and PKG serve as another source of crosstalk between the pathways. Both kinases share a number of substrates allowing for specific downstream events to be mediated by both cAMP and cGMP signaling [19]. For example, vasoactivator stimulated phosphoprotein (VASP) is preferentially phosphorylated at its serine 157 residue by PKA and its serine 239 residue by PKG in order to modulate focal adhesions, cell shape, or platelet aggregation in response to a number of stimuli [38]. Alternatively, PKA and PKG, while predominantly activated by their respective cyclic nucleotides, can, in some instances, be activated by the alternate cyclic nucleotide. For instance, high levels of cAMP have been found to activate PKG *in vitro* in the absence of cGMP, but the validity of this effect is questionable as it has not been observed in intact cells or under physiological conditions [29].

## 2. Cyclic Nucleotide Signaling in Cancer

As described in Table 2, altered expression and/or activity of one or more cyclic nucleotide signaling mediators have been reported in various carcinomas and hematological malignancies [39,40,41,42,43,44,45,46,47,48,49,50,51,52,53,54,55]. The role of cAMP and cGMP signaling and activation of their down-steam effectors (i.e*.*, PKA and PKG) is complex when determining their stimulatory and inhibitory actions in cancer cells. Both cAMP and cGMP signaling have been found to have either positive or negative effects on cell growth and survival, depending on cell or tissue type [40,41,42,43,44,45,46,56,57,58,59,60]. These observations, suggest that aberrant cyclic nucleotide signaling may play an important role in tumorigenesis.

**Table 2:** General overview of reported alterations of cyclic nucleotide signaling and down-stream effectors in select cancer types. Changes in expression and/or activity are indicated as; ↑ refers to increase, whereas ↓ refers to decrease.

Cancer Type	Observed Alteration	Reference
**Bladder**	↑ PDE5 expression	[40,61]
	↑ MRP5 expression	
**Breast**	↑ PDE expression and activity	[39,52,62,63,64]
	Altered PDE localization	
	↑ MRP5 expression	
**Colon**	↑ GC-C expression; ↓ ligand expression	[36,51,65]
	↓ PKG expression	
	↓ PKA expression	
**Hepatoma**	↑ basal levels of cAMP and cGMP	[53]
**Leukemia**	Altered PDE isozyme expression	[46,66]
	↑ PDE activity	
**Lung **	↑ PDE expression and activity	[61,67]
	↑ MRP5 expression	
**Lymphoma**	↑ PDE activity	[49,50]
	↓ basal levels of cAMP and cGMP	
**Ovarian**	↓ basal levels of cAMP	[54,61]
	↑ MRP5 expression	
**Pituitary**	↓ AC activity	[55]
	↑ PDE expression and activity	
**Prostate**	↑ MRP5 expression	[61]
**Skin**	↑ PDE activity	[67]

### 2.1. Cyclic Nucleotide Signaling in Hematological Malignancies

Numerous studies have found alterations in cyclic nucleotide signaling in hematological malignancies when compared to nonmalignant hematopoietic cells [46,49,50,66,68,69,70,71,72,73,74,75,76]. Predominantly, these alterations result in an attenuation of cAMP signaling with unaltered or amplified cGMP signaling. This may be due to selective PDE activity being as much as 10–20 fold higher in leukemia cells compared to normal lymphocytes [46]. Changes in the rate and capacity for cAMP hydrolysis suggest a decrease in the amplitude and/or duration of cAMP signals within these cells. While this effect may be due to an increased expression of nascent cAMP-degrading PDE enzymes, other studies suggest that it may be due to isozyme switching in which the malignant cells “turn off” the expression of certain forms of PDE while “turning on” the expression of other forms that are not normally expressed. For example, chronic lymphocytic leukemia (CLL) cells have been found to have decreased expression of the cAMP-degrading isozymes that are commonly expressed in lymphocytes under physiological conditions, PDE3B and 4D, with proportional increased expression of PDE7B, a PDE isozyme not expressed in healthy lymphocytes [66].

The relevance of altered cyclic nucleotide signaling, especially cAMP signaling, in hematological malignancies has been investigated extensively [20,46]. Most studies have concluded that cAMP signaling promotes differentiation of hematopoietic progenitor cells while inhibiting proliferation and inducing apoptosis in lymphocytes, an effect that appears to be independent of hematopoietic cell type [46,69,71]. Such effects suggest that decreasing the responsiveness of hematopoietic cells to cAMP signaling via increases in the expression of PDE enzymes or decreases in the expression of AC enzymes could instill a growth advantage and potentially promote tumorigenesis. Another explanation is the selective involvement of the PKA isozymes, CREB activation and the role of cAMP signaling on selective cancer cells [20,77]. Alternatively, cGMP signaling has been found to have minimal effects on differentiation, growth, and survival of hematopoietic cells [46,68], potentially explaining why cGMP signaling is less commonly altered in hematological malignancies compared to cAMP signaling.

### 2.2. Cyclic Nucleotide Signaling in Epithelial Tumors

Deregulation of cAMP and cGMP signaling and their down-stream effector pathways occurs during cancer development and progression. One of the most well characterized aberrations in cGMP signaling involves decreased expression of the peptide hormones, guanylin and uroguanylin, in colorectal adenomas and adenocarcinomas that is accompanied by an increased expression of the guanylin receptor, guanylyl cyclase C (GC-C) [78,79,80,81]. Consistent with this observation, colorectal cancer cells generally have decreased basal levels of cGMP signaling and are hypersensitive to cGMP signaling activation with GC-C ligands compared to the normal colonic mucosa [78,80,82]. Mammary tumors have also been found to possess altered cyclic nucleotide signaling, particularly in terms of hydrolytic capacity [62,63,67,83,84,85]. For example, faster growing and more invasive mammary tumors show an overall decreased ability to hydrolyze cyclic nucleotides [62,63,67]. While this may be due to alterations in PDE expression, there also appears to be an alteration in compartmentalization of PDE isozymes [62]. Alternatively, MRP5, which exports cyclic nucleotides, particularly cGMP, from cells and serves as a mechanism in addition to PDEs for termination of intracellular cGMP signaling, has been found to be overexpressed in a number of epithelial cancers including cancers of the lung, bladder, ovaries, prostate, breast, and colon [61,86].

The regulatory roles cGMP and cAMP have in different cell and tissue types can be utilized in cancer development in a specified manner. Involvement and activation of down-stream effectors such as, PKA, has been linked multiple cancer types [23]. Determining the different roles cGMP and cAMP play in cell survival or death is cancer and cell type specific. The oncogenic activity of cAMP in selective epithelial tumors is through the activation of PKA and down-stream effectors (i.e*.*, EPAC and CREB) [23,87]. In comparison, cGMP signaling appears to play an important role in promoting apoptosis and inhibiting proliferation of certain epithelial cells [65,80,88,89,90,91], suggesting that the decreased levels of cGMP signaling or the decreased responsiveness to cGMP signals that have been observed in a number of types of solid malignancies could instill a growth advantage and potentially promote tumorigenesis in these tissues.

While some have attributed the anti-proliferative and pro-apoptotic effects of cGMP signaling in epithelial cells to intracellular ion fluxes via activation of CNGs [92] and/or crosstalk with cAMP pathways via modulation of PDE activity [93], others have concluded that PKG activation is largely responsible for these effects [94,95,96]. In support of the latter possibility, studies have shown that expressing constitutively active forms of PKG enzymes results in increased apoptosis and decreased cell viability in colon cancer cell lines [36]. The three isoforms of PKG vary not only in their tissue expression but also in the intracellular proteins and pathways they regulate. For instance, PKGIα regulates several proteins including survivin, VASP, c-Src and surprisingly CREB [97]. PKGIα regulation of these proteins may be cell or tissue type specific, as activation of these proteins were demonstrated in either ovarian and/or lung cancer cells [97]. Demonstrating the difference in down-steam targets of the PKG isoforms, PKGIβ has been shown to regulate the phosphorylation of the androgen receptor and the cofactor p44 in prostate cancer cells [98]. In comparison, PKGII has been implicated in physiological functions including intestinal secretion, bone growth, learning and memory, apoptosis, and suppression of EGF signaling in breast cancer cells [99]. In addition to its profound effects on PKG activation and intracellular epithelial cell processes, cGMP signaling has also been implicated in extracellular remodeling, an important step in tumor invasion and metastasis. Namely, activation of GC-C in colon cancer cells has been shown to reduce secretion of matrix metalloproteinase 9 (MMP-9) in a cGMP-dependent manner [100].

## 3. Targeting Cyclic Nucleotide Signaling for the Prevention and/or Treatment of Cancer

Because alterations in cyclic nucleotide signaling are common to a number of cancer types, appear to occur early in the tumorigenic process, and correlate with stage and prognosis, these pathways could provide new molecular targets for cancer chemoprevention and/or chemotherapy. However, as is the case with any signaling pathway, pharmacologically targeting the cyclic nucleotide pathways offers a number of challenges, particularly concerning toxicity due to lack of specificity. For this reason, targeted approaches for different pathway components, specifically the cyclases, kinases, and PDEs, have been investigated with varying degrees of success.

### 3.1. Targeting Cyclases

Due to the roles of cAMP and cGMP as negative regulators of cell growth, pharmacological activation of AC or GC enzymes to increase basal levels of cyclic nucleotides may be a logical approach for inhibiting the growth and viability of various types of cancer cells. While histamine and prostaglandin are examples of endogenous activators of AC that have been used experimentally, the botanical compound forskolin is widely used as a pharmacological activator of AC both *in vitro* and *in vivo* [101]. Studies with forskolin have shown an increase in tumor cell caspase-mediated apoptosis and differentiation with a decrease in proliferation and viability in multiple types of cancer, suggesting the potential efficacy of AC activation for cancer treatment [102,103,104,105]. Unfortunately, the nonselective activation of AC enzymes caused by forskolin and its derivatives is associated with a multitude of effects that preclude its use for most indications due to the potential for toxicity [106,107,108].

The activation of AC by β-adrenergic receptors (β-AR), and their down-stream effects of increased cAMP levels and activation of PKA, has been of clinical importance due multiple epidemiologic studies demonstrating the association between β-blockers and breast cancer [109,110,111]. However, there is conflicting data showing β-AR stimulation to both inhibit and stimulate breast tumor growth, or β-AR signaling having an insignificant effect on breast tumor growth [109]. In an effort to identify the potential role the β-AR pathway plays in breast cancer, several factors have been taken into consideration. Two of the major factors that have to be considered in these studies are the hormonal status of the breast cancer and the selectivity of the β-blockers used (i.e*.*, β1/β2-AR nonselective or β1-AR selective) [109,110]. The use of β-blockers has also been investigated in other cancers including prostate cancer, ovarian cancer, non-small cell lung cancer, and melanoma [109,110,111,112]. At this time further studies are required to understand the relative effects of the β-AR pathway on cancer development and progression to strategize preventative and therapeutic approaches.

A number of GC activators are commonly used *in vitro* and *in vivo*. Among these are the NO donors and sGC activators, such as sodium nitroprusside (SNP), and peptide activators of pGC, such as guanylin, uroguanylin, and heat-stabile enterotoxin (ST_h_). While the potential efficacy of the NO donors (e.g., NO-NSAIDs) and sGC activators for cancer chemoprevention and chemotherapy has been well documented [64,113,114,115,116,117], their potential for toxicity has precluded their development for either chemoprevention or chemotherapy [118]. In addition, it is unclear if the effects of all NO donors are related to the release of NO, as alternate mechanisms such as increased membrane permeability may be involved [117,119]. Conversely, studies performed with the pGC activators, particularly the GC-C activators guanylin, uroguanylin, and ST_h_, have been promising. Several features of GC-C make it a very attractive target for gastrointestinal cancers, especially colon cancer. First, this enzyme is almost solely expressed in gastrointestinal tissues, meaning that systemic administration of a GC-C agonist would have limited effects, reducing its potential for toxicity [88]. Additionally, GC-C expression in gastrointestinal cancers is appreciably higher than surrounding normal tissues, and studies suggest that lower concentrations of GC-C agonists are necessary to activate cGMP signaling in the cancer cells [79]. This allows for a second level of specificity, targeting not only the gastrointestinal tissue but also the diseased portion of that tissue, again suggesting the possibility of reduced toxicity. *In vitro* and *in vivo* studies have shown promising toxicity profiles and significant efficacy for a number of natural and synthetic GC-C agonists, and there is on-going research to develop these agents for both the prevention and the treatment of gastrointestinal cancers [65,82,88,89].

### 3.2. Targeting Phosphodiesterases

The large numbers of distinct protein products that comprise the PDE superfamily make inhibiting cyclic nucleotide degradation a promising target for the development of novel anticancer agents. Individual PDE isozymes differ in tissue expression patterns, subcellular localization, regulatory properties, and sensitivity to inhibitors [28], suggesting the possibility for selective targeting of a single PDE isozyme in order to increase the specificity and reduce the toxicity of a given agent [120]. As such, numerous studies have found alterations in the activity and expression of specific PDE isozymes in various types of cancers. For example, our laboratory has shown that PDE5A isozymes are overexpressed in breast and colon tumor cells while the expression of other cGMP PDE isozymes is significantly decreased [64]. This implies that selective inhibition of PDE5 could result in growth inhibition of tumor cells due to their reliance on PDE5 for termination of pro-apoptotic cGMP signaling with minimal effects on normal cells due to their expression of other cGMP degrading PDE isozymes. Targeting PDE5 inhibition in cancer cells demonstrates increased PKG activation, decreased β-catenin and survivin expression levels [121]. The inhibition of PDE5 activity leads to the increase of cGMP levels and thus the activation of its down-stream signaling pathway and the induction of apoptosis. PDE5 overexpression has been made by other investigators in breast, colon, bladder, and lung cancers, PDE7B in leukemia, PDE1C in glioblastoma, and several PDE4 isoforms in lung cancer [40,41,64,66,67,121,122].

The selectivity and improved toxicity profile potentially offered by targeting certain PDEs is promising as an anticancer target due to its advanced stage of development as a result of its efficacy and utility as a target for other indications. PDE inhibitors have been developed as therapies for a number of pathologies including heart failure, asthma, erectile dysfunction, and pulmonary hypertension [91]. As such, today’s researchers possess a collection of diverse PDE inhibitors with different biochemical and pharmacological properties including varying degrees of isozyme selectivity and elucidated toxicities, which, as reviewed previously [46,67,123], has prompted the ongoing effort to identify PDE inhibitors with potential anticancer efficacy. However, understanding of the regulatory mechanisms and potential adaptations of cancer cells for targeting selective PDEs, such as up-regulation of alternative PDE isozymes, increased cAMP signaling and alternative signaling is required. An example of one of these potential outcomes is PKGIα activation of CREB in lung cancer [97]. Careful consideration of the role selective cyclic nucleotides play in certain cell and cancer types as well as, evaluation of alternative outcomes need to be explored when targeting PDE activity as a cancer chemoprevention or therapeutic strategy.

### 3.3. Targeting Kinases

Because kinases have been shown to be largely responsible for mediating the growth-inhibitory effects of cyclic nucleotide signaling, pharmacological activation of these proteins may provide another target for cancer prevention or therapy. Unfortunately, targeting PKA is not a feasible approach considering the potential for toxicity, which is comparable to that observed for activation of AC enzymes. However, investigators have recently studied the effects of PKA activators given in combination with other agents and observed significant efficacy and synergism for reducing the viability of leukemia cells [124]. While these studies are preliminary, this is a promising approach as it could potentially result in reduced toxicity by lowering the dosage of the PKA activator that would be necessary to observe an effect. It has also been observed that there is a difference in the ratio between PKA-RI and PKA-RII in select cancer types [125,126]. Although more studies need to be conducted to fully understand the difference in the regulatory role of these isoforms these studies provide insight into the means of developing safe and effective inhibitors.

While few direct pharmacological activators of PKG have been identified with the exception of cGMP analogs such as 8-bromo-cGMP, whose activity is limited by poor cell permeability and rapid degradation by PDE, numerous studies suggest the efficacy of activating PKG for the prevention or treatment of cancer. For example, ectopic expression of constitutively active PKG isoforms has been shown to induce cell cycle arrest, inhibit tumor cell migration, reduce angiogenesis, and promote anoikis in colon cancer cells [36,127,128,129]. Alternatively, studies utilizing small molecule PDE inhibitors such as exisulind to activate cGMP signaling have found that downstream activation of PKG is a necessary step for their pro-apoptotic and growth-inhibitory activity in cancer cells [36,37,130], further suggesting the potential efficacy of direct PKG activation. Identifying a potential mechanism for PKG induction of apoptosis has become of considerable interest for cancer chemoprevention. It has been demonstrated that activation of PKG through increased cGMP levels induces apoptosis in a number of colon and breast cancer cell lines [34,131]. In a recent study, PKG was demonstrated to activate the transcription factor forkhead box O (FOXO4) in colon cancer cells. Activation of FOXO4 led to its sequestering of β-catenin and inhibition of TCF/LEF activity [34]. FOXO transcription factors can induce cell death, thus it is interesting to speculate that activation of FOXO4 is a potential mechanism for PKG pro-apoptotic signaling. However, this mechanism should be carefully evaluated since FOXO transcription factors exert diverse effects on cellular functions including survival, stress resistance and detoxification [132]. Although these data support the development of selective PKG activators for the prevention and/or treatment of cancer deeper understanding of the cellular mechanisms regulated and potentially activated is required.

## 4. Conclusions

For the first time in the history of the Surveillance, Epidemiology, and End Results (SEER) Program of the National Cancer Institute (NCI), the time period from 1999 to 2006 demonstrated a significant improvement in the 5-year survival rate of cancer patients, up 17.5% from the 50.3% five-year survival observed in the time period from 1975 to 1977, suggesting that early detection, prevention, and therapeutic strategies are beginning to have a positive effect on patient outcome [133]. However, significant strides remain to be made as cancer is still the second leading cause of mortality in the United States. Data collected in recent years and summarized here strongly suggest that misregulated cyclic nucleotide signaling capacity offers a survival advantage for certain tumor types, potentially promoting tumor growth and development. The complexity and controversy surrounding the mechanisms of cAMP and cGMP signaling and their down-stream effects on cancer cells depends on the myriad of factors discussed in this review. The deeper understanding of cyclic nucleotide signaling, down-stream factors and off-target effects is critical for the development of cancer chemoprevention agents. This insight will allow for the development of safer and more efficacious drugs for both prevention and treatment of multiple types of cancer by targeting cyclic nucleotide signaling at the level of cyclic nucleotide synthesis, receptor activation, or cyclic nucleotide degradation.
